# Soil fungal alpha diversity is associated with grassland productivity across a natural aridity gradient

**DOI:** 10.3389/fmicb.2026.1888112

**Published:** 2026-09-03

**Authors:** Mingyi Shi, Chenghang Du, Yongbin Li, Niwu Te, Wentao Luo, Shaofeng Wang, Xiangfeng Zeng

**Affiliations:** 1 Key Laboratory of Industrial Ecology and Environmental Engineering (Ministry of Education, China), School of Environmental Science and Technology, Dalian University of Technology, Dalian, China; 2 Key Laboratory of Pollution Ecology and Environmental Engineering, Institute of Applied Ecology, Chinese Academy of Sciences, Shenyang, China

**Keywords:** aridity, climate change, community assembly, grasslands, primary productivity

## Abstract

Drylands are expanding as a consequence of ongoing climate change, yet the mechanisms through which soil microbiomes are associated with grassland productivity across aridity gradients remain unclear. Here, we characterized bacterial and fungal communities across four sites spanning a natural aridity gradient (from semi-humid to arid) in Inner Mongolia, encompassing meadow, typical, and desert steppes, to assess how microbial diversity, interaction networks, and assembly processes are related to above- and below-ground net primary productivity (NPP). Fungal phylogenetic diversity dropped notably with increasing aridity, decreasing from 200.09 at the semi-humid site to 86.03 at the arid site, representing a 57.0% reduction across the gradient, accompanied by reduced network complexity, intensified deterministic environmental filtering, and an enrichment of drought-tolerant taxa such as *Chytridiomycota* and *Glomeromycota*. Concurrently, ANPP and BNPP decreased from 357.99 ± 145.36 and 272.97 ± 70.44 g/m^2^ in meadow steppes to 12.08 ± 8.41 and 18.45 ± 12.75 g/m^2^ in the desert steppe, respectively. In contrast, bacterial diversity remained comparatively stable, although its network stability decreased and community assembly shifted from deterministic to stochastic processes in drier soils. Keystone taxa displayed clear turnover along the aridity gradient, and biomarker analysis identified distinct aridity-responsive bacterial and fungal groups. Fungal diversity, rather than bacterial diversity, showed the strongest positive association with both components of NPP, while aridity exerted strong indirect negative effects by reducing soil nutrients and fungal diversity. Overall, these findings demonstrate that soil fungal diversity is closely associated with grassland productivity under increasing drought conditions, emphasizing the necessity of conserving fungal diversity to maintain ecosystem functioning in a drying world.

## 1 Introduction

Grasslands cover approximately one-quarter of the Earth’s terrestrial surface and sustain essential ecosystem functions such as carbon sequestration, climate regulation, biodiversity maintenance, and livestock production (Bardgett et al., 2021; Sun et al., 2024; Wang et al., 2022). These ecosystems are highly sensitive to climate variability, particularly to shifts in precipitation patterns that are intensifying with global climate change (Burrell et al., 2020; Song L. et al., 2024; Wan et al., 2024). Increasing drought frequency and severity have triggered widespread degradation in grassland stability and productivity, with severe consequences for terrestrial carbon balance and regional food security (Liu et al., 2023; Ma et al., 2021). As a central indicator of ecosystem function, net primary productivity (NPP) responds strongly to water availability, though the magnitude and direction of change vary across grassland types and environmental contexts (Jochum et al., 2020; Liu et al., 2018; Shi et al., 2020). Understanding the biological and environmental determinants of NPP in drying landscapes is therefore essential for predicting grassland resilience under future climate scenarios.

Soil microbial communities are integral to nutrient cycling, plant growth, and ecosystem stability, making them key mediators of grassland responses to drought (Liu et al., 2023; Sokol et al., 2022). Microbial diversity and community composition influence plant nutrient acquisition, competitive dynamics, decomposition processes, and soil carbon turnover (Martins et al., 2024; Sokol et al., 2022; Wang et al., 2023). However, bacteria and fungi differ substantially in their drought tolerance strategies, ecological functions, and responses to environmental filtering (Martins et al., 2024). Bacteria often exhibit rapid turnover and physiological plasticity, whereas fungi typically possess extensive hyphal networks and spore-based dispersal mechanisms that can confer greater resistance to water stress (de Vries et al., 2018). These functional differences suggest that bacterial and fungal communities may contribute differently to ecosystem functioning under aridification (Chen et al., 2023; Li et al., 2021; Liang et al., 2019; Tao et al., 2023). On the Mongolian Plateau, ANPP and plant richness declined with aridity, while surface-soil fungal biomass and diversity showed negative linear relationships with aridity (Yang W. et al., 2023; Yang Y. et al., 2023). Despite these advances, the relative importance of bacterial versus fungal communities in mediating NPP along natural aridity gradients remains unclear, emphasizing the necessity of a comprehensive evaluation across the meadow–typical–desert steppe continuum.

Recent advances in microbial ecology have emphasized the importance of interaction networks, keystone taxa, and community assembly processes for predicting ecosystem dynamics under environmental stress (Banerjee et al., 2018; Jiang et al., 2024; Jiao et al., 2022b). Co-occurrence networks capture potential microbial interactions that underpin nutrient cycling and functional stability, while keystone taxa-though often low in abundance-can disproportionately shape community structure and ecosystem processes (Jiao et al., 2021; Qiao et al., 2024; Zhou et al., 2024). Similarly, deterministic and stochastic assembly processes shape microbial communities by filtering species according to environmental stress or neutral processes such as dispersal (Riddley et al., 2025; Xun et al., 2019). Although drought-induced shifts in microbial community composition and assembly mechanisms have been documented, it remains unclear how these changes cascade to influence grassland productivity across natural aridity gradients (Li et al., 2023; Oram et al., 2025).

Dryland ecosystems are projected to expand globally, making it increasingly critical to understand the mechanisms through which soil microbiomes regulate ecosystem functioning in water-limited environments (Huang et al., 2020; Maestre et al., 2021; Wu et al., 2023). Arid ecosystems are typified by strong environmental filtering, nutrient scarcity, and low soil organic matter, conditions that may intensify microbial dependence on drought-tolerant taxa and alter their contributions to carbon and nutrient cycling (Ochoa-Hueso et al., 2017). Despite the ecological importance of these processes, regionally explicit studies integrating microbial diversity, network complexity, assembly mechanisms, and plant productivity along natural aridity gradients remain scarce (Li M. et al., 2024). Existing studies often focus on individual microbial groups or controlled drought experiments, limiting the ability to generalize findings across landscape-scale environmental gradients (Li et al., 2025; Xiang et al., 2025).

The Inner Mongolia grassland transect spans a natural aridity gradient (mean annual precipitation: 175 mm to 362 mm; mean annual temperature: −2.4 °C to 5.6 °C), with grassland types transitioning from meadow steppe to desert steppe. This provides an ideal natural laboratory for investigating soil microbial responses to aridification and their impacts on ecosystem function (Miao et al., 2025). Using this spatial gradient, we combined high-throughput sequencing, co-occurrence network analysis, null-model inference, and structural equation modeling to assess microbiome restructuring under drought and its consequences for grassland productivity. Specifically, we examined (1) shifts in bacterial and fungal diversity, composition, and biomarker taxa; (2) changes in microbial interaction networks, keystone taxa, and assembly processes; and (3) the direct and indirect pathways through which aridity, soil properties, and microbiomes regulate above- and below-ground NPP. By linking microbial traits to ecosystem productivity, this work reveals how soil microbiomes mediate grassland functioning under increasing drought and provides a mechanistic basis for conserving microbial diversity to bolster the resilience of global drylands.

## 2 Materials and methods

### 2.1 Experimental design and sample collection

The study was conducted at four sites spanning an east–west aridity gradient in the arid and semi-arid grasslands of northern China. Although mean annual temperatures were similar among sites, mean annual precipitation decreased from east to west across meadow steppe (ET: 119°58′E, 49°19’N; HS: 119°30′E, 50°12’N), typical steppe (XY: 116°33′E, 43°32’N), and desert steppe (WL: 106°58′E, 41°25’N) (Luo et al., 2023; Ma et al., 2022). Detailed information of these study sites is provided in Supplementary Table S1. At each site, six plots (6 m × 6 m) were established in August 2020, and plant and soil samples were collected in August 2021. Plant biomass was oven-dried at 80 °C to constant weight. Soil cores (2.5-cm diameter, 0–10 cm depth) were taken after litter removal, and 200 g subsamples were sieved (2 mm) to remove roots and debris. Each soil sample was divided into two fractions: one was air-dried for physicochemical measurements and the other was stored at −80 °C for DNA extraction.

### 2.2 Meteorological data, soil properties and net primary productivity

The annual arid index (AI) data for China were sourced from the Loess Plateau Science Data Center^1^. This dataset was obtained based on the monthly precipitation (PRE) and potential evapotranspiration (PET) dataset of 1 km in China and calculated as the ratio of annual PET to annual PRE. The AI serves as an indicator of a region’s aridity or moisture level. Based on AI classification, regions can be categorized as humid (AI<1, typically corresponding to forest), semi-humid (1 ≤ AI < 1.5, corresponding to forest grassland), semi-arid (1.5 ≤ AI < 4, corresponding to dry grassland) and arid (AI≥4, corresponding to desert) (Peng, 2023). The four experimental sites had AI values of 1.50 (ET, semi-humid), 1.41 (HS, semi-humid), 2.21 (XY, semi-arid), and 6.53 (WL, arid), spanning a gradient from semi-humid to arid conditions.

We measured several key soil properties, including pH (potentiometrically; Zhou et al., 2020), bulk density (ring knife method; Yang W. et al., 2023), total phosphorus (perchloric-sulfuric acid digestion; Liu et al., 2023), available phosphorus (molybdenum-antimony anti-spectrophotometry), ammonium nitrogen and inorganic nitrogen (KCl extraction with flow analyzer; Zhou et al., 2020), and soil organic carbon and total nitrogen (elemental analyzer; Wang et al., 2024). At the end of growing season, we estimated ANPP by harvesting all aboveground biomass from two 0.5 × 0.5-m quadrats in each plot designated for destructive measurements at all sites (Song L. et al., 2024). Peak biomass was used as an indicator of annual ANPP, since plant growth in the study region is largely complete by this time. BNPP was measured using the root ingrowth core method (Persson, 1980). In early May 2021, two holes (5 cm in diameter) were drilled to 10 cm depth in each plot and refilled with root-free soil enclosed in nylon mesh (2 mm pore size). At the end of August 2021, soil cores were extracted. Roots therein were picked out by hand washing, oven-dried and weighed to achieve dry weight. BNPP was expressed as root dry weight per square meter in the 0–10 cm layer (Ma et al., 2022).

### 2.3 DNA extraction and high-throughput sequencing

Soil DNA was extracted with the Power Soil DNA Isolation Kit (MoBio). DNA quality and concentration were assessed on a NanoDrop 2000 spectrophotometer (Thermo Fisher Scientific, USA). The DNA was then submitted for commercial sequencing at Majorbio (Shanghai, China). The bacterial 16S rRNA gene (338F/806R) and fungal ITS region (ITS1F/ITS2R) were targeted for high-throughput sequencing on the Illumina MiSeq platform under a paired-end protocol (Burrell et al., 2020; Salas-Gonzalez et al., 2021).

Raw sequences were processed in QIIME2 for quality control and trimming. Chimeras were removed using DADA2 (Song B. et al., 2024). High-quality sequences were clustered into operational taxonomic units (OTUs) at 97% similarity with Usearch (v7.0.1090). To ensure comparability across samples, the OTU table was rarefied to 50,000 sequences per sample. OTUs with a total read count of less than 10 across all samples or present in fewer than three samples were removed to reduce the influence of rare and potentially spurious OTUs. The remaining OTU table was then converted to relative abundance for downstream analyses, including alpha diversity, community composition, and differential abundance analysis. Taxonomic classification was assigned against the SILVA database (v138) for bacteria and UNITE database (v8.0) for fungi. Sequences identified as chloroplasts, mitochondria, or those unclassified at the kingdom level were discarded from subsequent analysis. The obtained raw sequence data have been submitted to the NCBI database (accession no PRJNA1226389).

### 2.4 Statistical and bioinformatics analyses

Microbial diversity was characterized by *α*-diversity, including the Shannon index and phylogenetic diversity for both bacterial and fungal communities, while community structure was characterized by *β*-diversity (Bray–Curtis dissimilarity). All metrics were computed following established protocols (Li Y. et al., 2024; Oksanen et al., 2020). Differentially abundant genera between groups were identified using the Wilcoxon rank-sum test. Biomarker taxa associated with soil characteristics and key soil predictors of microbial indicators were both identified and evaluated using Random Forest models implemented with the randomForest package in R (Ren et al., 2024). Additionally, LEfSe was performed to identify biomarkers significantly enriched across sample groups from phylum to genus level (Segata et al., 2011).

Microbial co-occurrence networks were generated, representing significant biotic interactions via Spearman correlations (0.6 < |r| < 1, *p* < 0.05). Following construction, network properties were analyzed using the igraph R package for node and link quantification (Li Y. et al., 2024; Liu et al., 2026) and Gephi for topological and structural visualization (Bastian et al., 2009). Network complexity was assessed based on topological parameters (Jiao et al., 2022a; Li Y. et al., 2024), with hubs identified as nodes exhibiting high degree (>35 for bacteria, >15 for fungi) and closeness centrality (>0.3) (Agler et al., 2016; van der Heijden and Hartmann, 2016; Xiong et al., 2021). The average variation degree (AVD) was employed to evaluate community stability (Jiang et al., 2023).

Bacterial functional annotation was performed using FAPROTAX, while fungal trophic modes and ecological guilds were predicted using FUNGuild (Nguyen et al., 2016). A null model approach was applied to quantify the relative contributions of deterministic versus stochastic processes in microbiome assembly (Jiao et al., 2020; Zhou et al., 2014; Zhou and Ning, 2017). Biogeographic patterns were examined by calculating geographic distances with the geosphere package and assessing community similarity using the vegan package (Green and Bohannan, 2006). PLSPM analysis was employed to quantify the direct and indirect effects of aridity, soil properties, and biological factors on grassland productivity components (BNPP and ANPP) using the PLSPM package in R (Zhuang et al., 2024). Latent variables were defined as follows: aridity was represented by the aridity index; soil nutrients included total phosphorus, available phosphorus, total nitrogen, soil organic carbon, nitrate nitrogen, and ammonium nitrogen; soil physical properties included pH and bulk density; bacterial and fungal diversity were assessed by Shannon index and phylogenetic diversity; community stability was measured by average variation degree; keystone taxa were represented by the relative abundance of hub nodes; and productivity was represented by above-ground net primary productivity and below-ground net primary productivity. The goodness-of-fit (GOF) of the partial least squares path model was evaluated. Partial correlation analysis was performed using the ppcor package in R to evaluate the direct associations between fungal diversity and productivity while controlling for soil properties (pH, Bulk density, TP, AP, TN, SOC, NO₃^−^-N, and NH₄^+^-N). To assess the robustness of the PLS-PM results against site-specific effects, we performed a leave-one-site-out sensitivity analysis by sequentially excluding each of the four sites (ET, HS, XY, and WL) and re-estimating the model. All data analyses and figures generation were performed using Origin 9.5 or R software.

## 3 Results

### 3.1 Microbial community composition and diversity along the aridity gradient

**Figure 1:**
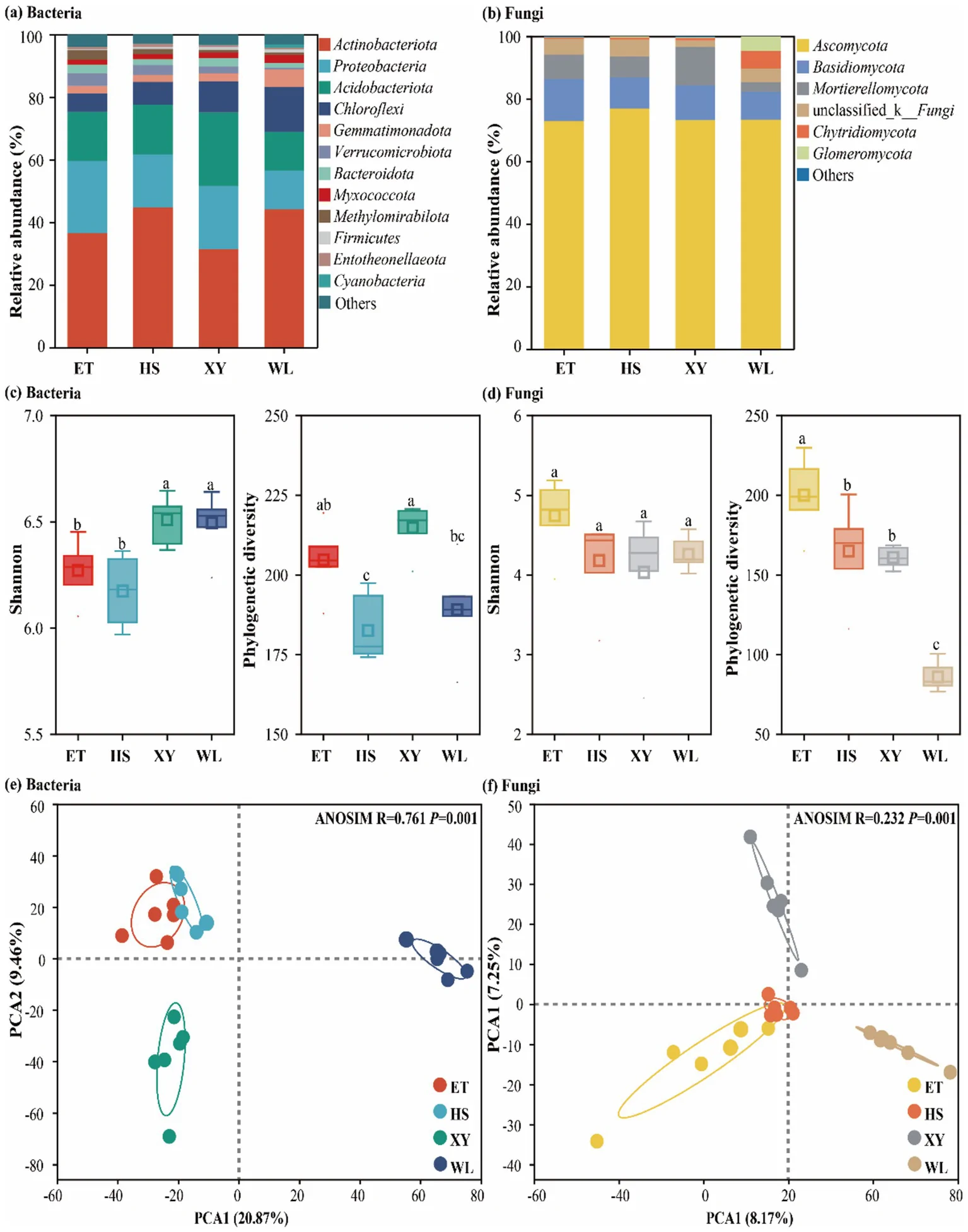
Bacterial and fungal domain phylum **(a,b)**, community diversity **(c,d)** and structure **(e,f)** in four regions. Different letters suggest significant differences (*p* < 0.05) by LSD test. ET, semi-humid; HS, semi-humid; XY, semi-arid; WL, arid.

For bacteria, *Actinobacteriota*, *Proteobacteria*, *Acidobacteriota*, and *Chloroflexi* accounted for 81.15–85.04% of sequences (Figure 1a). With increasing aridity, the relative abundance of *Chloroflexi*, *Gemmatimonadota*, *Myxococcota*, *Firmicutes*, and *Cyanobacteria* increased, whereas *Proteobacteria*, *Verrucomicrobiota*, and *Methylomirabilota* declined. For fungi, *Ascomycota*, *Basidiomycota*, and *Mortierellomycota* comprised 85.29–96.59%, with *Chytridiomycota* and *Glomeromycota* rising and *Basidiomycota* dropping under higher aridity (Figure 1b). Bacterial Shannon diversity was higher in WL and XY than in ET and HS (Figure 1c). In contrast, fungal phylogenetic diversity declined significantly along the aridity gradient (Figure 1d). PCoA showed clear separation of both bacterial and fungal communities among four regions (Figures 1e,f).

### 3.2 Biomarkers of significant enrichment in microbiomes along the aridity gradient

**Figure 2:**
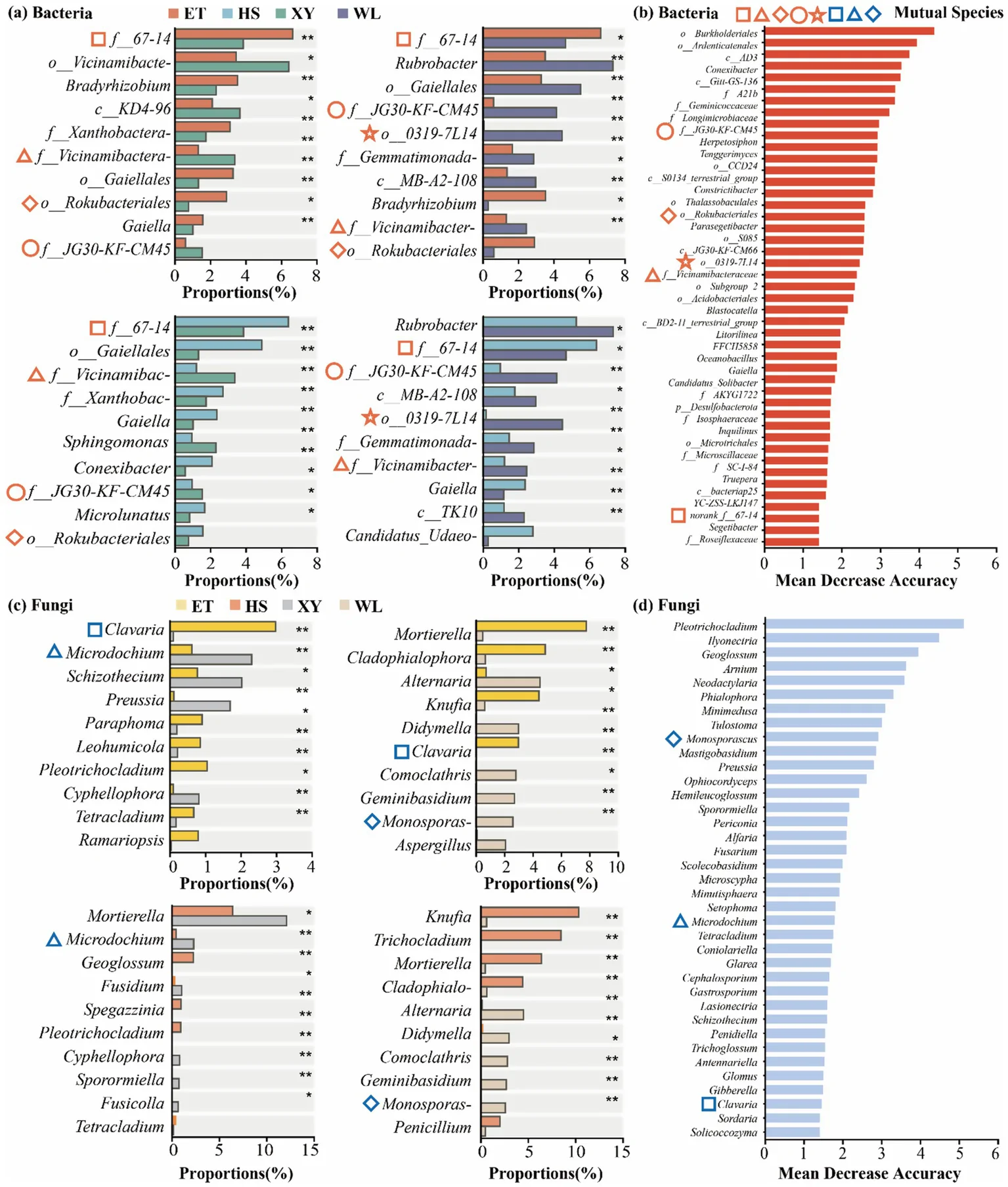
Phylotypes significantly different between four regions at genus level for bacteria **(a)** and fungi **(c)**. Data were shown as relative abundance (%) of genus in each group. Statistical analysis was performed by the Wilcoxon rank-sum test. *n* = 6, in each group. *p < 0.05, **p < 0.01. Ranking of the top 50 bacterial **(b)** and fungal **(d)** biomarkers by importance based on mean decrease accuracy in random forest selection. The abundance variation of the top 50 biomarkers across all samples. Mark mutual species using symbols.

LEfSe analysis identified bacterial and fungal taxa that were significantly enriched at the phylum to genus level in grassland soils across four arid regions (Supplementary Figure S2). The numbers of significantly enriched bacterial taxa in ET, HS, XY and WL were 18, 8, 11, and 21, respectively. For fungi, the corresponding numbers were 12, 13, 10, and 26 (Supplementary Figure S2). At the genus level, differences in bacterial and fungal abundances across arid regions were assessed using the Wilcoxon rank-sum test (Figures 2a,c). The bacterial genus *Rubrobacter* exhibited significant variation among four regions. For fungi, *Mortierella* abundance differed significantly between ET and WL, while *Knufia* varied between HS and WL.

Random forest analysis identified the key biomarkers predictive of regional aridity (Figures 2b–d). The top three bacterial biomarkers were *g_*unclassified_*o_Burkholderiales*, *g_*unclassified_*o_Ardenticatenales*, and *g_*unclassified*_c_AD3*. For fungi, the most important biomarkers were *Pleotrichocladium*, *Ilyonectria* and *Geoglossum*. In total, 72 bacterial OTUs and 21 fungal OTUs were characterized as key discriminators (Supplementary Tables S2, S3). Notably, five bacterial taxa were identified as shared across regions, including g_unclassified_*f_67–14*, *g_*unclassified _*f_Vicinamibacteraceae*, *g_*unclassified_*o_Rokubacteriales*, *g*_unclassified_*f_JG30-KF-CM45* and *g_*unclassified_*o_0319-7 L14* (Figures 2a,b and Supplementary Figure S2a). Among fungi, three shared taxa were identified as *Clavaria*, *Microdochium*, and *Monosporascus* (Figures 2c,d and Supplementary Figure S2b).

### 3.3 Complexity and stability of microbial interaction networks

**Figure 3:**
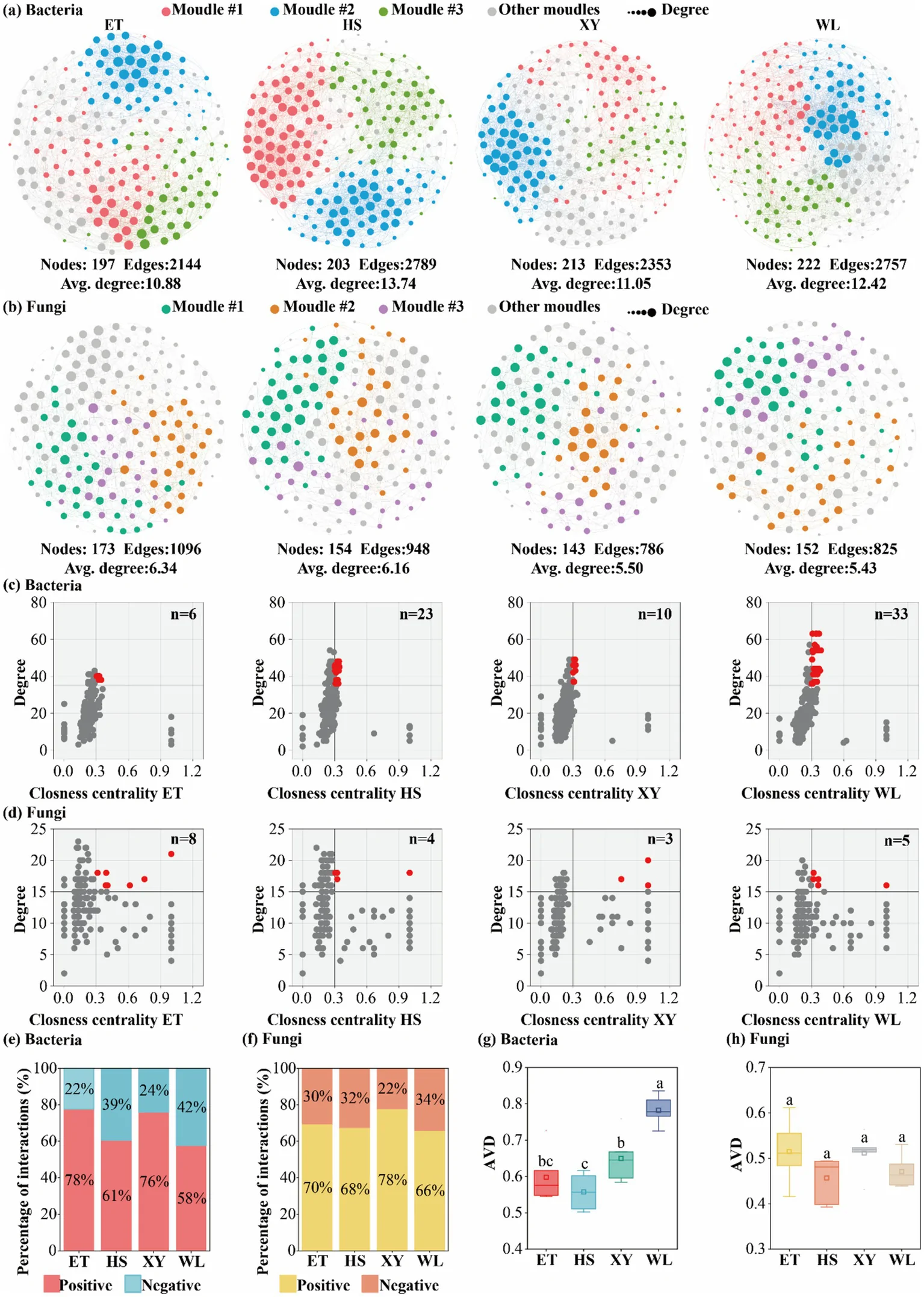
**(a,b)** Bacterial and fungal co-occurrence networks along the soil based on samples collected in 2021 (*n* = 24). Network diagram with nodes colored according to each of the four main ecological clusters. **(c)** Distribution patterns of the ‘hub nodes’ of bacterial network in different regions. **(d)** Distribution patterns of the ‘hub nodes’ of fungal network in different regions. **(e)** Bacterial co-occurrence network characteristics different regions. **(f)** Fungal co-occurrence network characteristics different regions. **(g,h)** Average variation degree of bacterial and fungal community. Different letters suggest significant differences (*P* < 0.05) by LSD test.

Analysis of co-occurrence networks revealed distinct responses of bacterial and fungal communities to aridity. Bacterial networks exhibited an increase in node number with drought severity, rising from 197 in ET to 222 in WL, and edges increased from 2,144 (ET) to 2,789 (HS). Average degree also varied, highest in HS (13.74) and lowest in ET (10.88) (Figure 3a, e, f and Supplementary Table S4). For fungal networks, ET showed the highest node and edge counts (173 and 1,096), whereas XY had the lowest (143 and 786). Average degree declined along the aridity gradient, from 6.34 in ET to 5.43 in WL (Figure 3e, f). These patterns indicate that fungal networks were more sensitive to aridity than bacterial counterparts, with drought markedly reducing fungal network complexity.

The network hubs were identified based on high degree values (bacteria >35, fungi >15) and closeness centrality (>0.3) within the network. A total of 14 network hubs (6 bacteria and 8 fungi) were observed in ET, 27 (23 bacteria and 4 fungi) in HS, 13 (10 bacteria and 3 fungi) in XY, and 38 (33 bacteria and 5 fungi) in WL (Figures 3c, d). The stability of the bacterial community, inferred from the AVD, decreased significantly (*p* < 0.05) with increasing aridity, following the trend WL > XY > ET > HS (Figure 3g). In contrast, fungal community stability showed no significant variation across the four aridity regions (Figure 3h).

### 3.4 Distribution and functional profiles of keystone taxa along the aridity gradient

**Figure 4:**
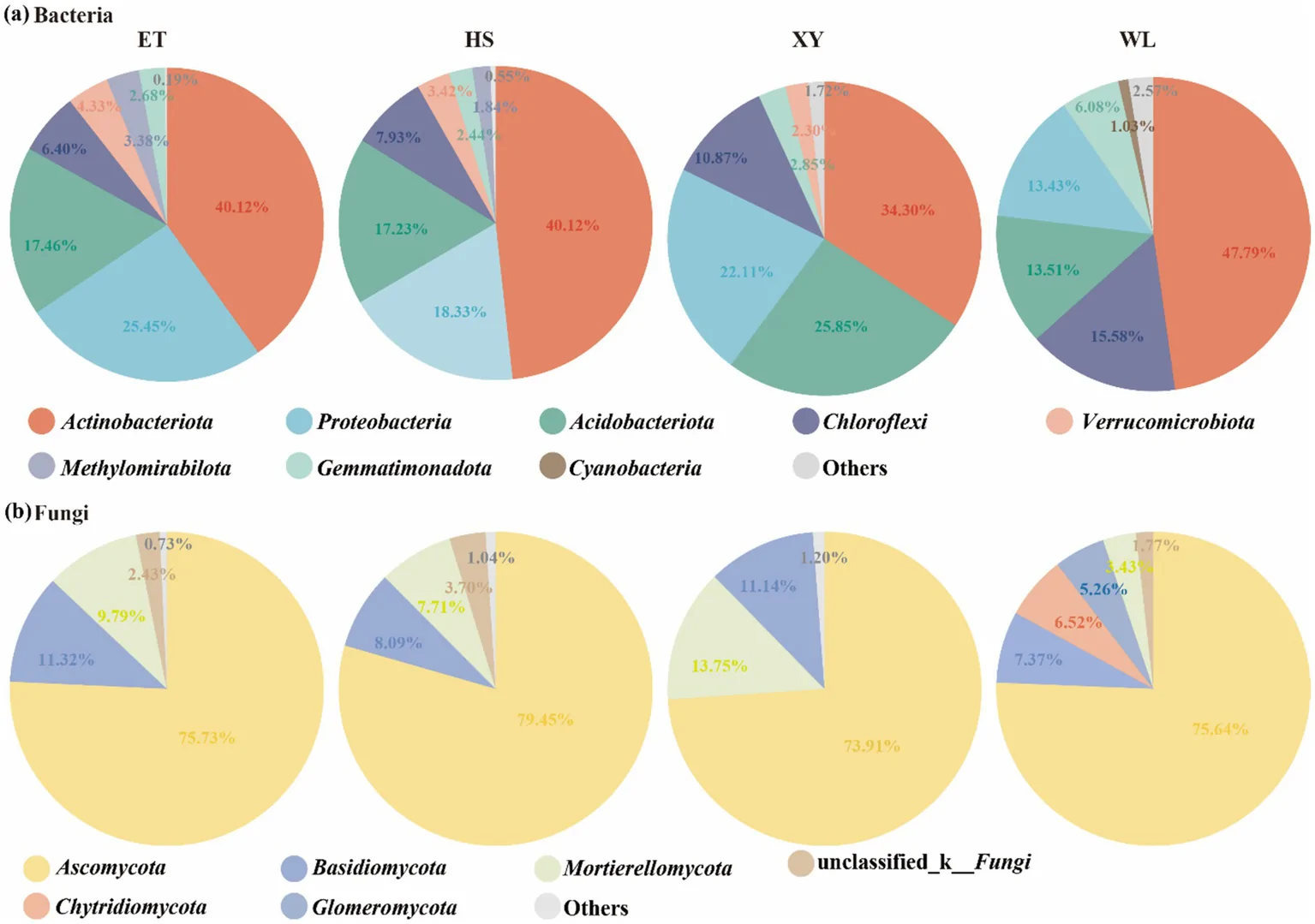
Community pie chart distribution of bacterial **(a)** and fungal **(b)** keystone taxa at the phylum level.

At the phylum level, the relative abundance of bacterial keystone *Chloroflexi* and *Gemmatimonadota* increased with rising aridity, while *Verrucomicrobiota* and *Methylomirabilota* decreased gradually (Figure 4a). Notably, *Cyanobacteria* was identified as a keystone phylum exclusively in the most arid region (WL), with an abundance of 1.03%. Conversely, *Methylomirabilota* was primarily found in the less arid regions, with relative abundances of 3.38% in ET and 1.84% in HS (Figure 4a). Among fungi, *Chytridiomycota* (6.52%) and *Glomeromycot* (5.26%) were identified as keystone phyla only in WL (Figure 4b). Additionally, the fungal keystone taxa became more diverse with greater aridity (Figure 4b).

The functional profiles of bacterial and fungal keystone taxa were consistent with those of the entire microbial community (Supplementary Figure S5). The predominant predicted functions of bacterial keystone taxa were chemoheterotrophy, aerobic chemoheterotrophy, and nitrate reduction. The abundance of phototrophic functions, including photoheterotrophy, phototrophy and photoautotrophy, increased with aridity. For fungal keystone taxa, the top three functions were participation in the synthesis of adenosinetriphosphatase, L-arabinose isomerase, and glucan 1,4-alpha-glucosidase. The functional abundance of fungal keystone taxa was significantly higher in regions with high aridity compared to those with low aridity.

### 3.5 Assembly processes and biogeographic patterns of bacterial and fungal communities

**Figure 5:**
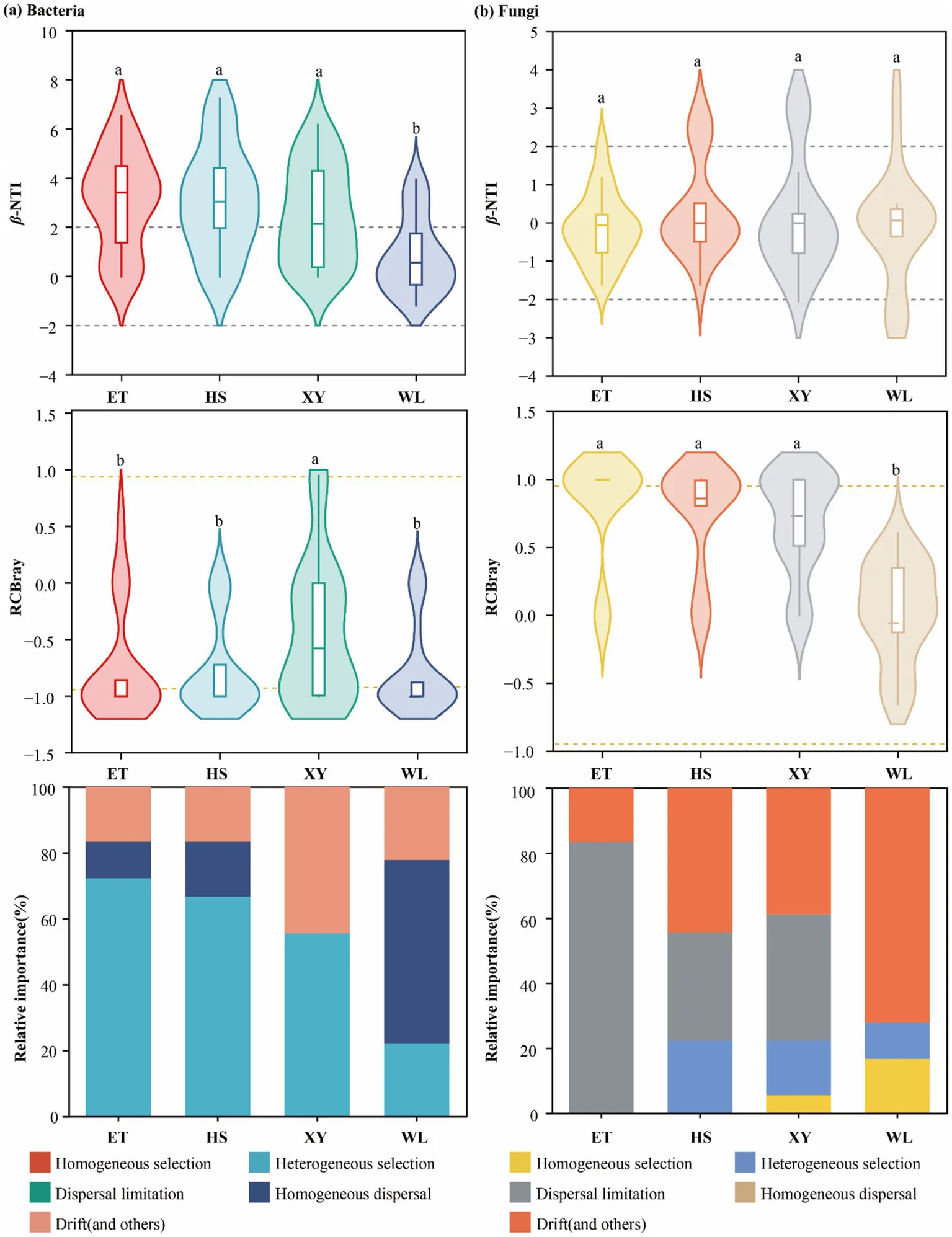
Variation in Beta Nearest Taxon Index (*β-NTI*) **(a,b)**, Bray–Curtis-based Raup-Crick (RCbray) indices **(c,d)**, and assembly process **(e,f)** of bacteria and fungi across four regions. *β-NTI* values were calculated based on weighted UniFrac distances. |*β-NTI*| > 2 representing the deterministic process, including heterogeneous selection and homogeneous selection. |*β*-NTI| < 2 represents stochastic processes, including dispersal limitation, homogeneous dispersal, drift and other processes. |*β*-NTI| < 2 represents stochastic processes, with *β*-NTI < 2, RCbray> 0.95 for dispersal limitation, RCbry<−0.95 for homogeneous dispersal, and | RCbray| < 0.95 for drift and other processes.

The contribution of ecological processes to the assembly of bacterial and fungal communities varied significantly across the aridity gradient (Figure 5). Integration of *β*-NTI (based on weighted UniFrac distances) and RCBray revealed distinct assembly mechanisms between bacterial and fungal communities along the aridity gradient. Bacterial *β*-NTI decreased progressively from 3.52 in ET and 3.30 in HS to 2.82 in XY and 0.96 in WL, while RCBray remained negative across all sites (ranging from −0.82 to −0.52), indicating a transition from deterministic processes (heterogeneous selection) in meadow steppes to stochastic processes (homogenizing dispersal) in the desert steppe, with XY representing an intermediate transitional state. In contrast, fungal *β*-NTI remained near zero across all sites (ranging from −0.11 to 0.24), whereas RCBray declined from 0.87 in ET and 0.85 in HS to 0.77 in XY and 0.06 in WL, suggesting that stochastic processes consistently governed fungal community assembly throughout the aridity gradient. Notably, with increasing aridity, bacterial community assembly exhibited a transition from deterministic to stochastic processes, whereas for fungi, the influence of deterministic processes gradually intensified.

The clear distance decay and incremental relationships between geographic distance and microbial communities are depicted in Supplementary Figure S6 (note that *β*-NTI values in Supplementary Figure S6d were calculated using unweighted UniFrac distances for robustness assessment). Soil aridity heterogeneity increased significantly with geographic distance (Supplementary Figure S6a). For bacterial communities, |ΔAVD| and *β*-NTI increased significantly as geographic distance expanded, while |Δphylogenetic diversity| decreased significantly. In contrast, for fungal communities, |ΔAVD| demonstrated a marked decline with increasing geographic distance, whereas |Δphylogenetic diversity| and *β*-NTI both increased noticeably (Supplementary Figures S6b–d).

### 3.6 Biotic and abiotic drivers of net primary productivity

**Figure 6:**
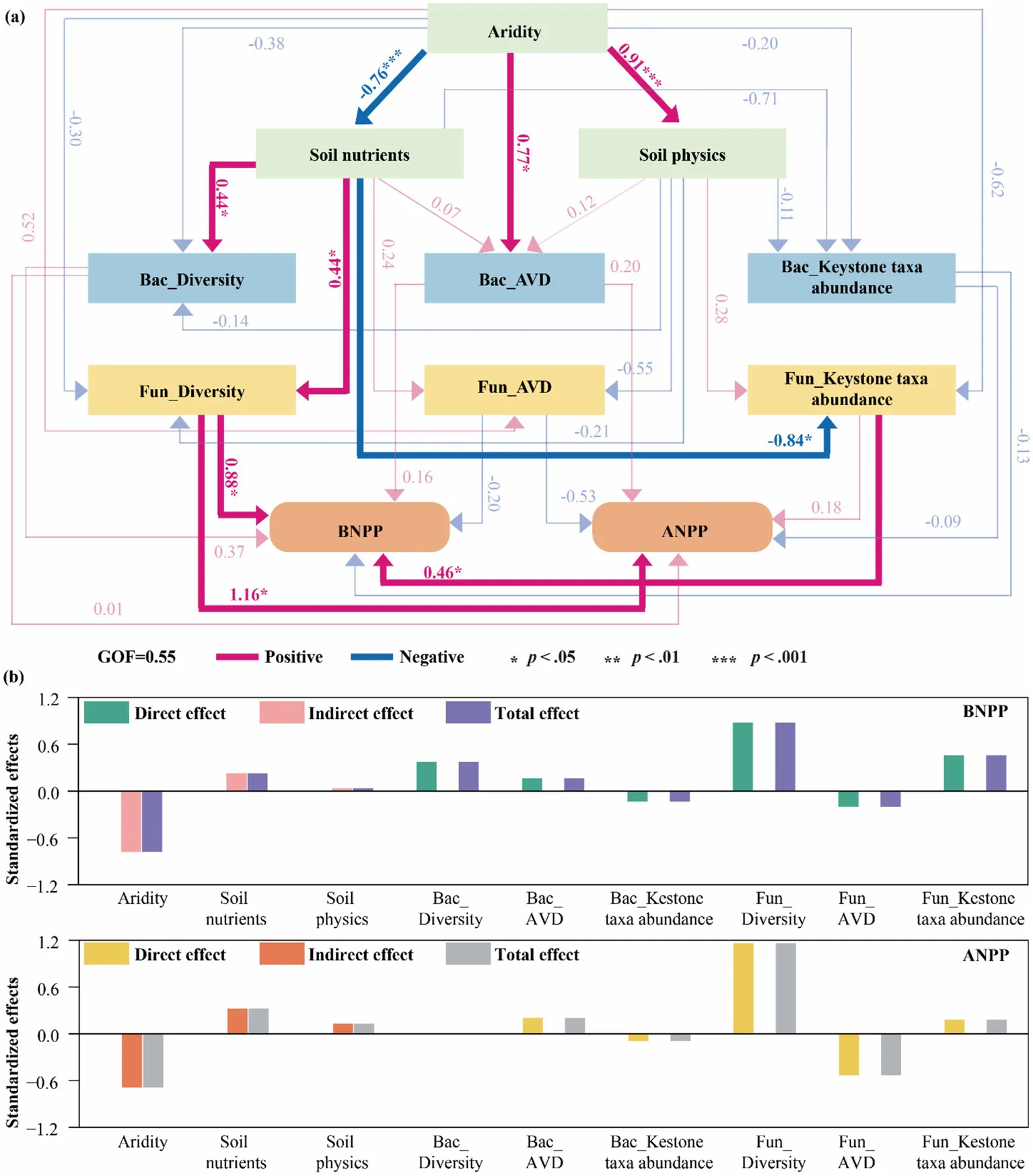
**(a)** Direct and indirect factors affecting BNPP and ANPP were determined using partial least squares path modeling (PLS-PM). Red and blue arrows indicate positive and negative effects, respectively, numbers above the arrows indicate standardized path coefficients. **(b)** The bar chart represents the standardized effects of PLS-PM on BNPP and ANPP.

Aridity influenced ANPP and BNPP through both direct and indirect pathways (Figure 6). Aridity was positively associated with soil physical variability and bacterial community variation, but negatively associated with soil nutrient availability. Soil nutrients, in turn, were positively associated with bacterial and fungal diversity, yet negatively associated with the abundance of fungal keystone taxa. Fungal diversity emerged as the strongest positive correlate of both ANPP and BNPP, whereas fungal keystone taxa were specifically linked to BNPP. Aridity was indirectly and positively associated with BNPP via its positive association with fungal keystone taxa abundance, and indirectly and negatively associated with both productivity components through its negative association with fungal diversity. Overall, the model identified aridity as the strongest negative correlate of both ANPP and BNPP, and fungal diversity as the strongest positive correlate.

We performed three complementary analyses to validate these findings. First, partial correlation analysis, controlling for soil physicochemical properties (pH, bulk density, TP, AP, TN, SOC, NO₃^−^-N, and NH₄^+^-N), revealed that fungal Shannon diversity remained significantly correlated with BNPP (partial *r* = −0.5299, *p* = 0.0348), but not with ANPP (partial *r* = −0.3349, *p* = 0.2048). Fungal phylogenetic diversity showed no significant correlations with either productivity component (*p* > 0.05) (Supplementary Figure S7). Second, leave-one-site-out sensitivity analysis, in which each of the four sites (ET, HS, XY, and WL) was sequentially excluded, demonstrated that the core path “fungal diversity → productivity” remained broadly consistent in direction across most subsamples, although its magnitude varied depending on the excluded site, and the direction reversed when either the most humid (ET) or the most arid (WL) site was removed (Supplementary Figure S8). Third, random forest analysis using PLS-PM-derived latent variables as response variables further identified the key soil predictors of microbial indicators (Supplementary Figure S9). Among the six microbial indicators examined, four models showed significant performance. For Fun_Diversity (*R*^2^ = 0.736, *p* = 0.001) and Bac_Diversity (*R*^2^ = 0.705, *p* = 0.001), pH, total phosphorus, soil organic carbon, total nitrogen, and bulk density consistently ranked as the top predictors. pH and total phosphorus were the primary predictors of bacterial diversity, whereas soil organic carbon and total phosphorus showed the highest importance for fungal diversity. For Bac_AVD (*R*^2^ = 0.539, *p* = 0.001), pH, total nitrogen, soil organic carbon, and total phosphorus were the main contributors. For Fun_Keystone taxa abundance (*R*^2^ = 0.236, *p* = 0.004), soil organic carbon, total phosphorus, total nitrogen, and pH were significant predictors.

Collectively, the PLS-PM, partial correlation, leave-one-site-out, and random forest analyses consistently identified soil pH and key nutrients (C, N, P) as primary predictors of microbial community structure, and revealed that the fungal diversity–productivity relationship was context-dependent.

## 4 Discussion

### 4.1 Aridity shifts the structure and characterization of soil microbiomes

Drought-induced nutrient limitation alters multiple attributes of soil microbial communities, including diversity, composition, and ecological roles (Berdugo et al., 2020; Peng et al., 2024). In our study, drought-induced nutrient limitation significantly altered soil microbial community structure and composition along the aridity gradient, as reflected by the compositional shifts at the phylum level (Figures 1a,b), the contrasting responses of bacterial and fungal *α*-diversity (Figures 1c,d), and the clear *β*-diversity turnover revealed by PCoA (Figures 1e,f). Fungal phylogenetic diversity declined sharply along the aridity gradient, decreasing from 200.09 in the semi-humid site (ET) to 86.03 in the arid site (WL)— a 57.0% reduction (Figure 1d). In contrast, bacterial Shannon diversity remained relatively stable across all sites (Figure 1c). This divergence may be attributed to the reductions in soil organic carbon availability under drought, which likely affect fungi more strongly than bacteria (Deng et al., 2021; Maestre et al., 2015). Elevated CO_2_ may partly buffer these impacts by enhancing plant water-use efficiency and subsequent carbon inputs to soil (Evans et al., 2014; Maestre et al., 2015). Beyond carbon limitation, declining soil moisture directly restructures microbial communities (Lennartz et al., 2023). Bacteria and fungi exhibit contrasting drought responses, which stem from fundamental differences in microbial strategies: bacteria rely on rapid physiological plasticity and stress-resistant traits such as endospore formation (Berendsen et al., 2016; Praveen Kumar et al., 2014), whereas fungi benefit from hyphal foraging networks (Rodriguez-Caballero et al., 2018), leading to divergent climate sensitivities (Barnard et al., 2013; de Vries et al., 2018).

Aridity strongly reshaped the ecological traits and taxonomic composition of soil microorganisms. *Proteobacteria* were more abundant in semi-humid and semi-arid soils, whereas drought-tolerant phyla such as *Chloroflexi* and *Firmicutes* dominated arid, nutrient-poor environments, consistent with their physiological resilience, spore formation, and thick cell walls that protect against osmotic stress (Chatoutsidou et al., 2023; Cui et al., 2023). Fungal communities were similarly reorganized, with *Ascomycota*, *Basidiomycota*, and *Mortierellomycota* as dominant taxa (Figure 1b). The increasing abundance of *Chytridiomycota and Glomeromycota* in drier sites suggests specific adaptations to water-limited conditions. The marked β-diversity turnover across the aridity gradient (Figures 1e,f) indicates enhanced vulnerability of soil microbiomes to climate-induced moisture decline in xeric regions, where reduced soil water retention constrains microbial metabolic activity (de Nijs et al., 2019; Ru et al., 2018).

Biomarker analysis identified strong drought-responsive taxa: drought-sensitive groups such as *Rubrobacter* and *Knufia* declined, whereas *Mortierella* increased with aridity. *Ascomycota*, including genera like *Monosporascus*, dominated under drought due to resistant traits or opportunistic strategies, including prolific spore production (Dong et al., 2024; Egidi et al., 2019; Lavallee et al., 2024; Maestre et al., 2015; Treseder et al., 2014). The increase of several bacterial (e.g., *Vicinamibacteraceae*, *JG30-KF-CM45*, *0319-7 L14*) and fungal (e.g., *Microdochium*) taxa suggests potential genetic or proteomic adaptations to water stress. However, the ecological mechanisms underpinning their functional contributions remain poorly understood, as do their potential feasibility and efficacy as microbial inoculants under drought. Therefore, identifying such biomarkers and keystone taxa is essential for predicting microbiome responses to climate extremes and for developing effective strategies to maintain soil ecosystem functions under increasing drought stress.

### 4.2 Aridity alters microbial network characterization and keystone species distribution

In our study, bacterial networks showed increased nodes (197 → 222) and edges (2,144 → 2,789) with aridity, whereas fungal networks exhibited reduced nodes (173 → 143) and average degree (6.34 → 5.43) (Figures 3a,b), indicating greater fungal sensitivity to drought. This result is consistent with established patterns wherein higher precipitation strengthens microbial networks, whereas desert soils harbor simpler and potentially more vulnerable communities (Wang et al., 2018). The observed increase in bacterial AVD indicates a significant loss of network stability, consistent with the notion that water stress disrupts established microbial interactions (de Vries et al., 2018; Pathan et al., 2023). These contrasting responses reflect both ecosystem-specific soil properties that influence microbial sensitivity to moisture change (Soleimani et al., 2019) and intrinsic differences in hydrological adaptation among microbial lineages (Wu et al., 2018). Overall, fungal networks were more sensitive to drought-induced stress, whereas bacterial networks showed greater structural destabilization under arid conditions.

Microbial groups that exhibit ecological dominance, biomarker signatures, or network centrality often function as keystone taxa, exerting disproportionate roles in structuring communities and driving ecosystem processes (Banerjee et al., 2018). Keystone taxa composition varied across regions (Figures 3c,d), with *Chloroflexi* and *Chytridiomycota* enriched in arid sites (consistent with phylum-level shifts in Figures 1a,b). Under intensified drought, *Chloroflexi* increased due to its physiological resilience and metabolic flexibility, allowing it to outcompete drought-sensitive bacteria. Among fungi, *Chytridiomycota* occurred exclusively in the most arid sites, reflecting its drought tolerance, low nutrient demands, and competitive advantage under reduced biotic pressure (Dacal et al., 2022). Identifying such dominant, diagnostic, and hub taxa provides a foundation for developing microbiome-based management strategies and effective microbial amendments (Agler et al., 2016; Qiu et al., 2019; Toju et al., 2018).

### 4.3 Factors driving microbial community assembly along the aridity gradient

Microbiome assembly is influenced by ecological determinants such as priority effects, where early colonizers shape subsequent community development (Carlström et al., 2019; Fukami, 2015). Our results demonstrate a clear divergence in these assembly rules between bacteria and fungi across the aridity gradient. Bacterial *β*-NTI decreased from 3.52 in ET and 3.30 in HS to 2.82 in XY and 0.96 in WL, while RCBray remained negative across all sites (−0.82 to −0.52), confirming a shift from deterministic to stochastic assembly with increasing aridity (Figure 5a). In contrast, fungal *β*-NTI remained near zero (−0.11 to 0.24), whereas RCBray declined from 0.87 in ET to 0.06 in WL (Figure 5b), indicating that stochastic processes consistently dominated fungal assembly, though deterministic filtering intensified under xeric conditions. The initial deterministic control on bacteria likely reflects their small cell size, rapid growth, and high metabolic flexibility, enabling efficient tracking of environmental change (Chen et al., 2021; Jiao et al., 2020; Zhang et al., 2021). Bacterial assembly was driven by precipitation, soil moisture, pH, SOC, and plant traits (Yang et al., 2022), with extreme pH strengthening deterministic selection and circumneutral pH promoting stochasticity (Tripathi et al., 2018).

Fungi show strong resilience to harsh environments due to traits such as spore formation and aerial dispersal (Roper et al., 2010). Increasing aridity intensified deterministic assembly in fungal communities (Figure 5d), suggesting stronger environmental filtering driven by climate-altered resource availability (Guo et al., 2018; Zhou et al., 2023). While drought enhances stochastic processes in protists (Chen et al., 2022; Zhang et al., 2022), rehydration can reduce fungal stochasticity in the rhizosphere. These patterns highlight the sensitivity of microbial assembly to external disturbances and the need to maintain ecosystem stability under environmental change (Wu et al., 2018). Environmental filters fundamentally structure microbial communities (Fang et al., 2023; Fu et al., 2024). As r-strategists, bacteria can rapidly respond to environmental shifts (Sun et al., 2017), whereas fungi, as K-strategists, exhibit slower growth but superior resource use efficiency (Yang Y. et al., 2023), potentially strengthening neutral processes relative to deterministic selection.

### 4.4 Linkages between aridity, soil microbiomes, and grass productivity

Aridity correlates with reduced grassland productivity, reflecting the high climate sensitivity of primary production in these ecosystems (Wu et al., 2021). As an integrative indicator of climate-soil-microbe interactions, the aridity index captures shifts that destabilize ecosystem functioning (Berdugo et al., 2020; Hu et al., 2021). Our results showed that aridity was significantly associated with ANPP and BNPP through both direct and indirect pathways (Figure 6). Aridity was positively associated with soil physical variability and bacterial variation, but negatively associated with soil nutrient availability. Soil nutrients were positively correlated with bacterial and fungal diversity, yet negatively correlated with key fungal taxa abundance. Fungal diversity emerged as the strongest positive correlate of both productivity components, whereas key fungal taxa specifically were linked to BNPP. Aridity was indirectly and positively associated with BNPP through its positive association with fungal keystone taxa abundance, but was indirectly and negatively associated with both productivity components via its negative association with fungal diversity. These findings highlight that fungal diversity, rather than bacterial diversity, exhibits the strongest association with grassland productivity under drought, consistent with the role of fungi in regulating biodiversity-productivity relationships through improved resource use efficiency and plant interactions (Chen et al., 2020; van der Heijden et al., 2008; van der Putten et al., 2016). Niche differentiation, saprotrophic mineralization (Bardgett and van der Putten, 2014), and ectomycorrhizal keystone taxa (Fahey et al., 2023) collectively support plant coexistence and productivity.

To further evaluate these associations, we performed three complementary analyses. Partial correlation analysis, controlling for soil physicochemical properties, confirmed that fungal Shannon diversity remained significantly correlated with BNPP (partial *r* = −0.5299, *p* = 0.0348), indicating that the observed relationship was independent of variation in soil physicochemical properties (Supplementary Figure S7). Leave-one-site-out sensitivity analysis revealed that the core relationship between fungal diversity and productivity remained generally consistent across subsamples, but reversed when either the most humid (ET) or the most arid (WL) site was excluded, suggesting that the association depended on environmental context rather than being driven by a single sampling site (Supplementary Figure S8). Random forest analysis further identified pH, total phosphorus, soil organic carbon, total nitrogen, and bulk density as the primary predictors of microbial diversity, reinforcing the central role of soil properties in shaping microbial community structure (Supplementary Figure S9).

The direction of the fungal diversity–productivity association differed between PLS-PM and partial correlation. This discrepancy likely reflects differences in the analytical frameworks. PLS-PM captures the total effect within a network of interacting variables, whereas partial correlation isolates the direct residual association after controlling for covariates. The negative direct association may suggest resource competition, while the positive total effect is likely mediated by indirect pathways via fungal keystone taxa. Furthermore, the context-dependent nature of this relationship, as revealed by the leave-one-site-out analysis, implies that the fungal diversity–productivity association is not a universal ecological law but rather a pattern shaped by environmental context, particularly position along the aridity gradient. Given the absence of plant community data, we cannot exclude the possibility that the observed relationship partially reflects plant community turnover along the gradient.

Beyond the specific associations between fungal diversity and productivity, the underlying microbial assembly processes also have important implications for ecosystem functioning. Diversity-driven deterministic assembly plays a central role in microbial carbon and nitrogen cycling, processes fundamental to plant growth and productivity (Xun et al., 2019). Ongoing climate change and human activities have posed serious threats to soil microbial diversity, with direct implications for plant performance (Classen et al., 2015; Maeder et al., 2002). Drought, a major constraint on productivity, is expected to intensify, yet its effects on root-associated microbiomes remain insufficiently understood, despite the drought-mitigating potential of specific microbial consortia (Xu et al., 2018). Plant-associated microbiomes across soil, rhizosphere, and roots enhance host stress resilience through dynamic interactions (Naylor and Coleman-Derr, 2017; Singh et al., 2020; Trivedi et al., 2022). Clarifying these mechanisms is essential for developing climate-resilient crops and sustainable agroecosystems.

### 4.5 Limitations of the study

While our study provides valuable insights into the spatial patterns of soil microbiomes along an aridity gradient, several limitations must be explicitly acknowledged. First, our findings are based on a single-year and single-season sampling event. This design is robust for describing spatial correlative patterns at a specific time point, but it inherently restricts our ability to evaluate interannual variability, seasonal microbial dynamics, and long-term ecosystem responses to chronic drought. Additionally, the precipitation and temperature at the study sites during the sampling period generally reflect typical local climatic conditions rather than those of years with extreme weather (Supplementary Table S1). Second, microbial analyses were conducted only for the shallow soil layer. Deeper soil layers may harbor distinct microbial communities with different sensitivities to water deficits, which warrants future investigation. Finally, plant community data, such as plant richness, vegetation cover, and dominant species composition, were not available for inclusion in our current analytical framework. Along the aridity gradient, plant communities inevitably undergo turnover, which influences both soil microbiomes and ecosystem productivity. Therefore, the positive relationship observed between fungal diversity and productivity may partially reflect plant community turnover rather than an independent effect of fungal diversity alone. Consequently, our findings should be interpreted strictly as spatial correlative associations rather than long-term mechanistic dynamics. Future studies incorporating multi-year, multi-season, and deep-profile sampling, alongside comprehensive vegetation surveys, are essential to fully unravel the long-term resilience of dryland ecosystems under future drying climates.

## 5 Conclusion

This study shows that increasing aridity is strongly associated with fundamental shifts in soil microbial communities and their relationships with grassland productivity. Fungal communities, particularly their diversity and keystone taxa, were far more sensitive to drought than bacterial communities and exhibited strong positive associations with both ANPP and BNPP. In contrast, bacterial diversity remained relatively stable, despite shifts in network stability and assembly processes. Our structural equation analysis indicated that losses of fungal diversity are closely associated with aridity-induced constraints on ecosystem productivity. Overall, this work highlights that soil fungal communities are closely linked to dryland ecosystem functioning and resilience, providing a basis for understanding grassland responses to ongoing climate change and for considering microbiome-based strategies to mitigate its impacts.

## Data Availability

The data analyzed in this study is subject to the following licenses/restrictions: the obtained raw sequence data have been submitted to the NCBI database (accession no PRJNA1226389). Requests to access these datasets should be directed to https://www.ncbi.nlm.nih.gov/.

## Footnotes

1
http://loess.geodata.cn
