# Supplementary material for: Soil fungal alpha diversity is associated with grassland productivity across a natural aridity gradient
Source: Front Microbiol. 2026 Sep 3;17:1888112. doi: 10.3389/fmicb.2026.1888112 (PMC13582366; doi:10.3389/fmicb.2026.1888112)
Supplement: Supplementary file 1 [file Data_Sheet_1.docx]

**Supplementary Figure 1.** Soil physicochemical properties

**Supplementary Figure 2.** Indicator bacteria(a) and fungal(b) with LDA scores of 4 or greater in bacterial and fungal communities associated with soil from the four regions. Mark mutual species using symbols.

**Supplementary Figure 3.** Network of co-occurring 90% cutoff OTUs based on correlation analysis. A connection stands for a strong (Spearman's *ρ*>0.6) and significant (*P*-value <0.05) correlation. The size of each node is proportional to the number of connections (that is, degree). Light green lines represent a significant negative correlation and light red lines represent a significant positive. OTUs colored by phylum taxonomy.

**Supplementary Figure 4.** Community pie chart distribution of bacterial(a) and fungal(b) keystone taxa at the genus level.

**Supplementary Figure 5.** Bacterial functional groups predicted by FAPROTAX (a) and fungal trophic modes and ecological guilds assigned by FUNGuild (b) across different degradation levels. After Z-score normalization, different data values are represented by the intensity of the colors in the heatmap.

**Supplementary Figure 6.** The relationships between geographic distance and bacterial and fungal community similarity. (a)The relationship between geographic distance and |ΔAI|. (b) The relationship between geographic distance and |ΔPhylogenetic diversity|. (c) The relationship between geographic distance and |ΔAVD|. (d)The relationship between *β*-NTI and geographic distance. *β*-NTI values in panel (d) were calculated based on unweighted UniFrac distances for robustness assessment.

**Supplementary Figure 7.** Partial correlation matrix of fungal diversity, productivity, and soil properties (pH, Bulk density, TP, AP, TN, SOC, NO₃⁻-N, and NH₄⁺-N) after controlling for soil covariates. Values are partial correlation coefficients; asterisks indicate significance (* p < 0.05, ** p < 0.01, *** p < 0.001). Blue and red represent negative and positive correlations, respectively.

**Supplementary Figure 8.** Leave-one-site-out sensitivity analysis of the PLS-PM model for the core path “fungal diversity → productivity”. Panels A–D show the path diagrams after sequentially excluding (A) ET (meadow steppe; GOF = 0.7394), (B) HS (meadow steppe; GOF = 0.6238), (C) XY (typical steppe; GOF = 0.6271), and (D) WL (desert steppe; GOF = 0.4276). Path coefficients are shown next to the arrows, with positive paths in red and negative paths in blue. Asterisks indicate significance (* p < 0.05, ** p < 0.01, *** p < 0.001). GOF = Goodness-of-Fit. Bac_Diversity and Fun_Diversity are composite latent variables extracted via PLS-PM from Bac_Shannon and Bac_Phylogenetic_diversity, and from Fun_Shannon and Fun_Phylogenetic_diversity, respectively.

**Supplementary Figure 9.** Variable importance of soil factors for microbial indicators based on random forest analysis. Panels A–D display the %IncMSE (% increase in mean squared error) of soil predictors for (A) Bac_Diversity (a composite latent variable extracted from Bac_Shannon and Bac_Phylogenetic diversity using PLS-PM), (B) Fun_Diversity (a composite latent variable extracted from Fun_Shannon and Fun_Phylogenetic diversity using PLS-PM), (C) Bac_AVD, and (D) Fun_Keystone taxa abundance. Asterisks denote significance levels (* 𝑝<0.05, ** 𝑝<0.01, *** 𝑝<0.001); ns indicates non-significant predictors. The random forest models were constructed using 500 regression trees with a sample size of 24.
